# Comprehensive investigations of biobutanol production by a non-acetone and 1,3-propanediol generating *Clostridium* strain from glycerol and polysaccharides

**DOI:** 10.1186/s13068-016-0641-8

**Published:** 2016-10-18

**Authors:** Fengxue Xin, Chao Wang, Weiliang Dong, Wenming Zhang, Hao Wu, Jiangfeng Ma, Min Jiang

**Affiliations:** 1 State Key Laboratory of Materials-Oriented Chemical Engineering, College of Biotechnology and Pharmaceutical Engineering, Nanjing Tech University, Puzhu South Road 30#, Nanjing, 211816 People’s Republic of China; 2 Jiangsu National Synergetic Innovation Center for Advanced Materials (SICAM), Nanjing Tech University, Nanjing, 211816 People’s Republic of China

**Keywords:** *Clostridium pasteurianum*, Biobutanol, Extractant, Elimination, By-products, Glycerol, Polysaccharides, Consolidated bioprocessing

## Abstract

**Background:**

Low-cost feedstocks, a single product (butanol), and a high butanol titer are three key points for establishing a sustainable and economically viable process for biological butanol production. Here, we comprehensively investigated the butanol production from mono-substrates, mainly glycerol and polysaccharides, mainly starch and xylan by a newly identified wild-type *Clostridium pasteurianum* GL11.

**Results:**

Strain GL11 produced 14.7 g/L of butanol with a yield of 0.41 g/g from glycerol in the batch mode without formation of by-products of acetone and 1,3-propanediol (1,3-PDO). With in situ extraction with biodiesel, the amount of butanol was finally improved to 28.8 g/L in the fed-batch mode. Genomic and enzymatic analysis showed that the deficiency of key enzymes involved in acetone and 1,3-PDO pathway within strain GL11 led to the elimination of these by-products, which may also greatly simplify downstream separation. The elimination of acetone and 1,3-PDO and high butanol tolerance contributed to its high butanol production yield from glycerol. More importantly, strain GL11 could directly convert polysaccharides, such as xylan and starch to butanol with secretion of xylanase and amylase via consolidated bioprocessing.

**Conclusions:**

The wild-type strain GL11 was found to be particularly advantageous due to its capability of efficient butanol production from glycerol and polysaccharides with elimination of acetone and 1,3-PDO formation. And the high butanol production with in situ extraction by using biodiesel would significantly enhance the economic feasibility of fermentative production of butanol from glycerol. These unique features of *C. pasteurianum* GL11 open the door to the possibility of cost-effective biofuels production in large scale.

## Background

Butanol, a four-carbon primary alcohol, is not only an important bulk chemical, but also considered as a promising next-generation liquid fuel because of its superior characteristics over ethanol [1, 2]. During the early twentieth century, butanol was industrially produced through the acetone–butanol–ethanol (ABE) fermentation from monosaccharides by *Clostridium acetobutylicum*. However, it was rapidly replaced by the petroleum-based butanol due to the loss of economical competitiveness [3]. Currently, there has been renewed interest in bio-based butanol production due to the depletion of fossil fuels and environmental concerns. However, sustainable large-scale biobutanol production is still impeded by three major hurdles: (1) high cost of traditional feedstocks: molasses, as typically used as a substrate in the past, may account for about 50 % of the final cost of the product; (2) low butanol titer due to the limited bacterial tolerance; and (3) high product recovery costs caused by the low production of butanol and the existence of by-products, such as acetone [1, 4].

One of the available strategies for circumvention of high-substrate costs is usage of inexpensive and abundant organic wastes as the fermentative substrates. Glycerol has recently been attracting much attention as a good substrate for bio-based production of chemicals, fuels, and materials as it is produced as a major by-product of biodiesel industry [5]. Due to the worldwide increment in biodiesel production, surplus glycerol is being produced annually [6]. Disposal of surplus glycerol has become a financial liability for biodiesel industries, which resulted in a significant decrease of the market price of glycerol. For example, in USA, the price of glycerol has decreased from US $0.20/lb in 2001 to US $0.01/lb in 2006 [7]. Its abundance and cost competitiveness make glycerol an excellent alternative to other carbon substrates for butanol production. Unfortunately, the most studied bacteria for biobutanol production, such as *C. acetobutylicum* and *C. beijerinckii*, can not grow solely in glycerol as it can not re-oxidize the excess NADH generated in the cellular glycerol catabolism [8, 9]. Currently, *C. pasteurianum* is the only solventogenic *Clostridium* species which possess the metabolic pathway needed for glycerol metabolism. However, butanol production by fermentation of *C. pasteurianum* from glycerol has been limited by the relatively low butanol titer as compared to those achieved by other sugar-based ABE fermentation processes, such as glucose or molasses. Meanwhile, the existence of 1,3-propanediol (1,3-PDO), which is another main product when using glycerol as the substrate, would reduce the overall butanol yield and further increase the subsequent cost of separation [10]. These make the identification of novel bacterial strains for specific applications, such as high butanol production from glycerol with elimination of other products as a promising prospect of future research.

Lipophilic butanol leads to low butanol titer, which in turn contributes to the high cost of product recovery [1]. Therefore, in situ solvent extractive fermentation has been proposed as one of the approaches to minimize butanol inhibition and increase product titer [11]. However, the market value of the extractant and the subsequent cost of extractant recycling have prevented them from being applied in large scale. An ideal in situ extractant should be the one that has a direct end-use as a fuel, which will bypass the expensive butanol recovery and extractant recycling procedure [12]. Biodiesel could serve as an excellent extractant for butanol production, as studies have shown that the ABE-enriched biodiesel obtained from the extractive fermentation possesses higher quality, such as the higher octane number (increased from 48 to 54) and the colder filter plugging point (decreased from 5.8 to 0.2 °C) [13]. Hence, development of glycerol-based butanol production process with in situ extraction using biodiesel can further add significant value to the biodiesel industry and also presents excellent potential to bring economy to the industrial production of butanol.

Currently, the most ideal solution for sustainable biobutanol production was consolidated bioprocessing (CBP) strategy using polysaccharides, such as lignocellulosic waste or cassava as the substrate, wherein microbes are used to hydrolyze and ferment inexpensive lignocellulosic materials directly into butanol without supplementation of hydrolytic enzymes [14]. Since the ability of butanol-producing bacteria to utilize cellulose/hemicellulose/starch is limited, an expensive hydrolysis step is required before fermentation to degrade the cellulose/hemicellulose/starch into simpler sugars. Although several metabolically engineered strains have been reported to generate value-added products directly from cellulose [15–17], however, no wild-type strains are known to produce butanol directly from cellulose or xylan, leaving a need for development of one-step strategy for biobutanol production from lignocellulosic materials.

Based on these, the main aims of this study are to firstly isolate and characterize novel butanol-generating microbes with capability of utilization of glycerol and polysaccharides, such as hemicellulose and starch, and also analyze the metabolic pathway via genomic sequence and comparison to elaborate its uniqueness. Then, further studies are carried out to improve final butanol titer via in situ extraction using biodiesel.

## Results

### Phylogenetic identification, genome sequencing and annotation of *C. pasteurianum* GL11

**Fig. 1:**
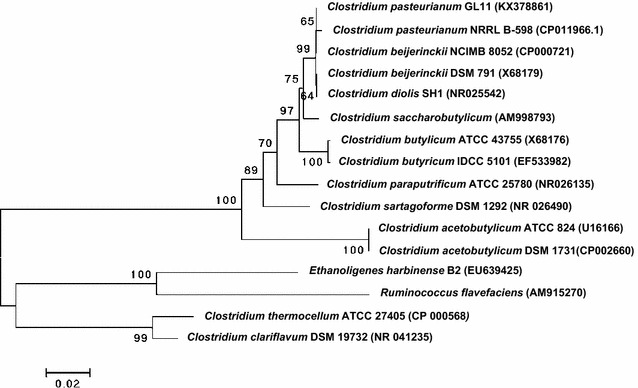
Phylogenetic tree of *C. pasteurianum* GL11 using the neighbor joining method (MEGA 4.0) based on 16S rRNA gene sequences

When xylan was adopted as the substrate, a colony (strain GL11) with relatively high xylanase activity was identified on agar plates after Congo red staining. When strain GL11 was further cultivated in mineral salts medium containing glycerol as the sole carbon source, the main metabolic products detected by GC-FID and HPLC were ethanol, butanol, and VFAs (acetate and butyrate), suggesting that strain GL11 synthesizes butanol via a unique pathway (butanol–ethanol, BE). The 16S rRNA gene sequence of strain GL11 with GenBank No. of KX 378861 shows 99 % identity to that of *C. pasteurianum* NRRL B-598 (NCBI Accession Number CP011966.1). Hence, it was designated as *C. pasteurianum* GL11. A phylogenetic tree based on 16S rRNA gene sequences was further established to show the relationship of the known *Clostridium* species (Fig. 1).

To better understand the genomic information and elaborate the butanol formation pathway, the whole genome of *C. pasteurianum* GL11 was sequenced and annotated by using a high-throughput sequencing whole-genome shotgun strategy (DDBJ/ENA/GenBank Accession No. MCGV00000000). Sequencing the genome of *C. pasteurianum* GL11 generated 77 large contigs ranging from 4226 to 4,485,768 bases. The sequence results showed that strain GL11 only contained chromosomal DNA and no plasmids were detected. GL11 consisted of an approximate 6.0-Mbp chromosome with a GC content of 30 %, which is much higher than that of *C. pasteurianum* DSM 525 (4.4-Mbp) and closely related to that of *C. pasteurianum* NRRL B-598 (6.2-Mbp) (Table 1). All the contigs of GL11 were predicted to possess 4319 coding sequences (CDSs), which is higher than that from *C. pasteurianum* NRRL B-598 and lower than that from *C. pasteurianum* DSM 525, 76 tRNA, and 21 rRNA sequences. The comparison between available genomic data from different *C. pasteurianum* strains is shown in Table 1.

**Table 1:** Comparison of genomic data from different *C. pasteurianum* strains

Feature	*C. pasteurianum* NRRL B-598	*C. pasteurianum* DSM 525	*C. pasteurianum* GL11
Genome size (bp)	6,186,879	4,350,673	5,993,599
GC content (%)	29.8	30	30
Protein-coding genes (CDS)	5002	3220	4319
rRNAs	49	10	21
tRNAs	94	81	76

### Preference of glycerol rather than glucose and xylose for butanol production by *C. pasteurianum* GL11 via a unique BE fermentation pathway

**Fig. 2:**
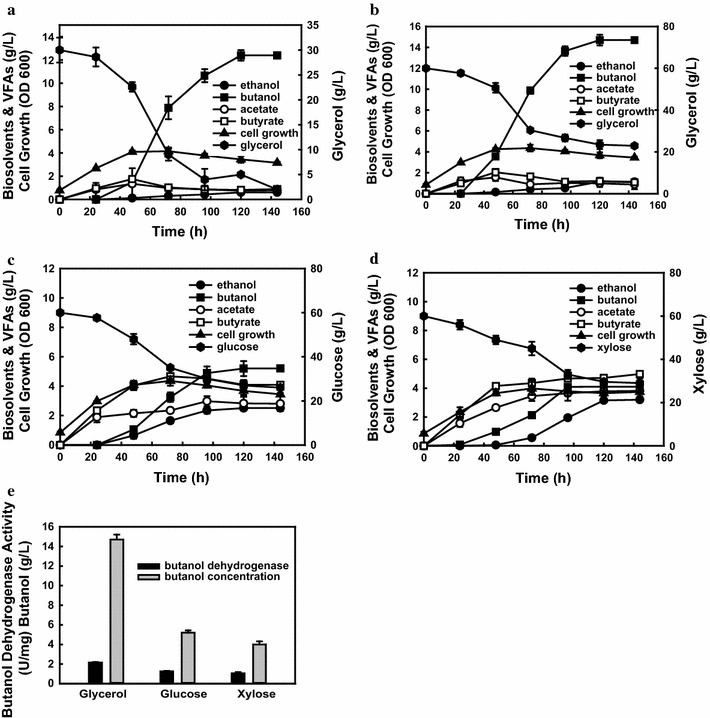
Growth and fermentation profiles of *C. pasteurianum* GL11 in P2 medium containing 30 g/L of glycerol (**a**), 60 g/L of glycerol (**b**), 60 g/L of glucose (**c**), 60 g/L of xylose (**d**) and butanol dehydrogenase activities (**e**). The pHs in the fermentation process were controlled above 5.2

*C. acetobutylicum* ATCC 824 and *C. beijerinckii* NCIMB 8052 are the two most well-known butanol-producing species, however, they showed limited glycerol utilization for butanol production. Currently, *C. pasteurianum* is the only characterized solventogenic species for efficient conversion of glycerol to butanol [5]. To investigate the butanol production potential by wild-type *C. pasteurianum* GL11, batch experiments were firstly conducted in mineral salts medium spiked with 30 g/L of glycerol with pH adjustment. As shown in Fig. 2, a classical butanol fermentation pattern was evident, with a first stage of acids production accompanying with the decrement of pH values (6.2–4.5) and fast cell growth (OD_600_ = 3.5) during the first 24 h, followed by a second stage of solvent formation with reutilization of parts of acids after pH was adjusted back to 5.5. After 122 h of fermentation, strain GL11 could produce 0.61 g/L of ethanol and 12.4 g/L of butanol, with negligible amount of acetone formation and glycerol leftover (Fig. 2a), suggesting a unique fermentative pathway for butanol production with elimination of acetone. More importantly, different from other *C. pasteurianum* strains, no 1,3-PDO production was detected in the medium broth when using glycerol as the substrate. The elimination of by-products (acetone and 1,3-PDO) contributes to a high butanol yield of 0.41 g/g. To further find the potential of butanol production by strain GL11, 60 g/L of glycerol was dosed into the medium. As shown in Fig. 2b, butanol titer could be further improved to 14.7 with 1.1 g/L of ethanol with a butanol ratio in total solvents of 93 %, which is much higher as compared to that (60 %) in the typical ABE fermentation process, indicating that strain GL11 is a promising candidate for converting waste glycerol into butanol.

It is known that glucose and xylose are the most typical hexose and pentose for ABE fermentation, which are also the main components in lignocellulosic hydrolysates [1, 2]. However, few studies regarding comparison of butanol production from glycerol, glucose, and xylose were carried out by using *C. pasteurianum* strains. Hence, butanol production from glucose and xylose was further evaluated and compared with that from glycerol (Fig. 2c, d). Unfortunately, strain GL11 shows much lower butanol production capability when glucose or xylose was used as the sole carbon source compared to that with glycerol as the substrate under similar conditions (60 g/L). Different from the typical bi-phase butanol fermentation pathway, strain GL11 shows high acids production no matter in glucose or xylose-amended medium. After 122 h of fermentation, strain GL11 could only produce 5.0 g/L of butanol and 2.5 g/L of ethanol in glucose-amended medium and 4.0 g/L of butanol and 3.2 g/L of ethanol in xylose-amended medium with no acetone production. These results indicate that glycerol is the most preferable substrate for butanol production within *C. pasteurianum* GL11 and it synthesizes butanol via a common metabolic pathway no matter in glycerol, glucose, or xylose, which is BE fermentation process. By further comparison of butanol dehydrogenase activities (BDH) in glycerol, glucose, and xylose, it was found that higher butanol production correlated with higher BDH activities, further suggesting that glycerol would be more preferable than glucose and xylose for butanol production by strain GL11 (Fig. 2e).

### High butanol tolerance in glycerol by *C. pasteurianum* GL11

To further investigate the mechanisms for high butanol production from glycerol, the influence of various concentrations of butanol on the bacterial growth and solvent formation was investigated. Culture GL11 was subjected to various levels of butanol challenge (5–18 g/L) during mid-exponential growth (OD_600_ = 1.0), and the growth after the addition of butanol was further monitored. It was found that culture GL11 can tolerate up to 15 g/L of butanol with no further butanol production and could barely survive at 18 g/L butanol in glycerol. With supplementation of 5, 10, and 15 g/L of butanol in 60 g/L of glycerol, the inhibition of cell growth is 19.4, 37.9, and 91.4 %, whereas culture GL11 was still able to produce 7.37 and 1.95 g/L of butanol with addition of 5 and 10 g/L of butanol, though a 20-h lag phase appeared for both setups. For both cultures, the butanol production persisted for 96 h and reached a total concentration of 12–13 g/L. As compared to the control (without the addition of exogenous butanol), cultures spiked with 5 g/L butanol produced 15.9 % less butanol, while cultures spiked with 10 g/L butanol produced 18.7 % less. The above observations indicated that culture GL11 was able to sustain higher amount of butanol (e.g., up to 15 g/L) than previously reported wild-type cultures (7.4–11.1 g/L) [1–3]. The resistance against high butanol suggests that strain GL11 may have the potential to be operated at higher butanol concentrations in an industrial chemostat.

### High butanol production from glycerol with in situ extraction using biodiesel

**Fig. 3:**
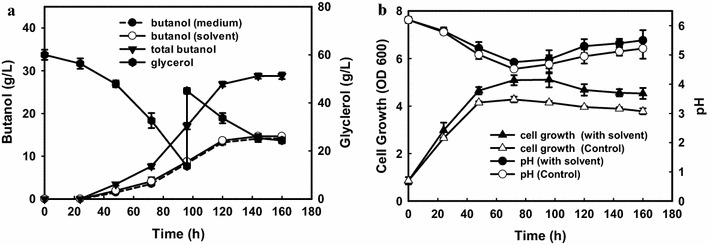
**a** Time course of butanol production and glycerol utilization in the fed-batch fermentation with the addition of biodiesel (ratio of 1:1); **b** time course of growth and pH in the batch fermentation with the addition of biodiesel (ratio of 1:1) and control batch (without addition of biodiesel)

It is well known that in situ removal of toxic solvent could minimize butanol inhibition and hence improve butanol titer [11]. Biodiesel can serve as a preferential extractant for butanol, which has a high partition coefficient for butanol (1.04) and especially, no toxicity to the clostridial cells. Hence, in order to further improve final butanol titer from glycerol, a fed-batch fermentation using biodiesel as in situ extractant with a volume ratio of 1:1 was examined (Fig. 3). As shown in Fig. 3a, 60 g/L of initial glycerol was rapidly consumed during the exponential phase after 96 h and 17.1 g/L of total butanol occurred with 8.33 and 8.72 g/L in medium and solvent phases separately, which corresponds to 16.3 % higher than the control (14.7 g/L) without addition of biodiesel (Fig. 2b). When another 30 g/L of glycerol was fed into the medium, butanol production could be further improved to 28.8 g/L with 14.5 of glycerol leftover (14.5 g/L) after 160 h of fermentation (Fig. 3a). 14.68 g/L and 14.15 g/L of butanol occurred in the fermentation broth and biodiesel phases, respectively (Fig. 3a). The simultaneous extraction of butanol could lead to higher amount of biomass (OD = 5.1 for 1:1; OD = 4.2 for the control) than the control, which may also contribute to the high butanol production (Fig. 3b). During the late solventogenic phase, there were only small amounts of ethanol (1.98 g/L), butyrate (0.33 g/L), and acetate (0.12 g/L) in the fermentation broth. Biodiesel has a partition coefficient of 1.2 for butyric acid, 1.1 for acetic acid, and 0.15 for ethanol, so only partial acids (0.13 g/L for butyric acid; 0.06 g/L for acetic acid) were transmitted from the fermentation medium to the solvent phase, which also resulted in a slightly higher pH value in the batch with extractant of biodiesel than that of the control batch (Fig. 3b). So, the butanol-enriched biodiesel with negligible ethanol and VFAs could be directly applied to the biofuel sector without the subsequent extractant recycling. Considering this, the biobutanol and biodiesel production plants can be designed in the same site in the future, where the by-products of glycerol from biodiesel plant can be directly used as the substrate for biobutanol fermentation; meanwhile, the enriched biodiesel which was adopted as the extractant for butanol production can be directly used in the fuel sector.

**Fig. 4:**
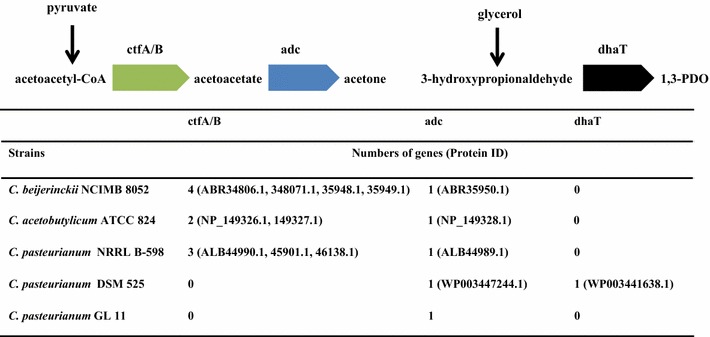
Arrangement of acetone and 1,3-PDO metabolic pathways with thin different solventogenic *Clostridium* strains

### Genomic analysis of metabolic pathway for solvent production within *C. pasteurianum* GL11

Typically, butanol production by solventogenic *Clostridium* strains in monosaccharides, such as glucose or xylose, is usually coupled with the formation of acetone or its derivative iso-propanol. However, the acetone-uncoupled production characterized in *C. pasteurianum* GL11 suggests that its acetone pathway may be deficient or blocked. Acetoacetyl-CoA transferase (ctfA/B) and acetoacetate decarboxylase (adc) are the two key enzymes in the acetone formation pathway, catalyzing the conversion of acetoacetyl-CoA, to acetoacetate and acetone [18] (Fig. 4). *C. acetobutylicum* ATCC 824 possesses two ctfA/B and one adc genes for acetone synthesis located in the plasmid (GenBank Accession No.:NC_001988.2), while *C. beijerinckii* NCIMB 8052 has four ctfA/B and one adc genes in the chromosome (GenBank Accession No.: CP000721.1). However, by the genomic annotation and analysis of *C. pasteurianum* GL11, it was found that ctfA/B gene was lacked in the whole genome and only one adc gene existed in the chromosome, implying that the deficiency of key ctfA/B gene is responsible for the elimination of acetone.

Currently, only two whole genomic sequences of *C. pasteurianum* strains are available, which are from *C. pasteurianum* DSM 525 (ATCC 6013) and *C. pasteurianum* NRRL B-598 [19, 20]. *C. pasteurianum* DSM 525 is the most widely used one for butanol production from glycerol as it only produced ethanol and butanol without acetone formation due to the deficiency of ctfA/B gene, which is consistent with our finding that the deficiency of ctfA/B gene in strain GL11 would lead to the inexistence of acetone (GenBank Accession No.: CP009268.1) (Fig. 4) [20]. However, high amount of 1,3-PDO occurred accompanying with butanol formation (Table 2). It is known that 1,3-PDO is synthesized by the catalysis of glycerol dehydratase and 1,3-propanediol dehydrogenase (dhaT) with consumption of NADH [21], the latter of which is the key enzyme in 1,3-PDO pathway from glycerol, catalyzing the conversion of intermediate of 3-hydroxypropionaldehyde to 1,3-PDO. One dhaT was found in *C. pasteurianum* DSM 525 (WP_003441638.1), which would take responsibility for 1,3-PDO synthesis. More recently, another whole genome from non-type *C. pasteurianum* NRRL B-598 has been sequenced (GenBank Accession No.: CP011966.1). Unlike other *C. pasteurianum* stains, strain NRRL B-598 showed inability to utilize glycerol and produced butanol and acetone as its main products with negligible formation of ethanol and 1,3-PDO [22]. Further genomic analysis showed that three ctfA/B and one adc genes existed in the chromosome, and no dhaT gene was found in its genome, explaining the formation of acetone and elimination of 1,3-PDO [19, 22]. With the genome annotation and analysis of strain GL11, it was found that there was no dhaT in the genomes of *C. pasteurianum* GL11. These are also consistent with the findings that no dhaT in the chromosome or plasmid of *C. acetobutylicum* ATCC 824 and *C. beijerinckii* NCIMB 8052 was found, suggesting that the deficiency of dhaT gene would result in the elimination of 1,3-PDO [23]. Therefore, the lackage of key genes for both acetone and 1,3-PDO formation in the whole genomes of *C. pasteurianum* GL11 would lead to the loss of acetoacetyl-CoA transferase and 1,3-PDO dehydrogenase activity, and thus attribute to its non-typical BE fermentation pathway.

**Table 2:** Comparison of solvent production by different solventogenic *Clostridium* species

Strain	Carbon source	1,3-PDO (g/L)	Ethanol (g/L)	Butanol (g/L)	Butanol ratio of total solvent (%)	References
*C. acetobutylicum* 2018adc^a^	Glucose (60 g/L)	n.a	2.8	13.6	82	[18]
*C. pasteurianum* DSM 525	Glycerol (91 g/L)	6.6	0.6	10	39.4	[24]
*C. pasteurianum* MBEL_GLY2^b^	Glycerol (86 g/L)	4.6	0.5	13.7	72.9	[24]
*C. pasteurianum* DSM525 (M2)^c^	Glycerol (46 g/L)	7.25	n.a	8.72	54.6	[9]
*C. pasteurianum* GL11	Glycerol (60 g/L)	n.a	1.1	14.7	93.0	This study

^a^ *C. acetobutylicum* 2018adc: The *adc*-disrupted mutant of *C. acetobutylicum* EA 2018; the medium supplemented with 1 % calcium carbonate and 6 mg/L methyl viologen [18]

^b^ *C. pasteurianum* MBEL_GLY: Mutant obtained from *C. pasteurianum* DSM525 after mutagenesis using NTG (*N*-*methyl*-*N*-*nitro*-*N*-*nitrosoguanidine*) [24]

^c^ *C. pasteurianum* DSM525 (M2): Mutant obtained from *C. pasteurianum* DSM525 after mutagenesis using ENU (*N*-*ethyl*-*N*-*nitrosourea*) [9]

### Direct conversion of polysaccharides—starch and xylan for butanol production by *C. pasteurianum* GL11

**Fig. 5:**
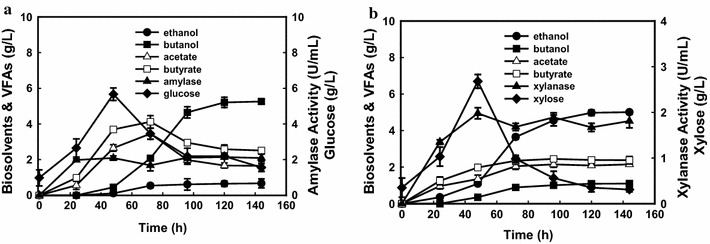
Metabolic profiles and enzymatic activities by *C. pasteurianum* GL11 when amended with 60 g/L of starch (**a**) or birchwood xylan (**b**) at 35 °C

Although solventogenic *Clostridium* species utilize a broad range of monosaccharides, disaccharides, and other substrates, such as inulin, pectin, and whey, they cannot directly utilize cellulose and hemicellulose for butanol production [2]. With the catalysis of lignocellulose-degrading enzymes, such as cellulase and xylanase et al., the solventogenic *Clostridium* could ferment the component sugars (glucose and xylose) in the lignocellulosic hydrolysate to biobutanol. Genome sequence analysis of *C. pasteurianum* GL11 showed the presence of polysaccharides-degrading enzymes (cellulase, xylanase, and amylase); therefore, direct fermentation strategies for butanol production were subsequently investigated by using *C. pasteurianum* GL11 in the presence of 60 g/L cellulose, xylan, and starch without supplementation of exogenous enzymes (Fig. 5). Unfortunately, strain GL11 did not show any growth in cellulose, although it contained some cellulose-degrading genes due to complicated assembly and expression of functional mini-cellulosomes in *Clostridium* strains [1, 2]. However, it could efficiently hydrolyze starch and xylan via secretion of related degrading enzymes for butanol and ethanol production. Under 60 g/L of starch-amended medium, strain GL11 was capable of efficiently secreting amylase (1.95 U/mL) and leading up to 5.62 g/L of accumulated glucose during the first 48 h. Meanwhile, strain GL11 could directly convert the glucose to a final butanol of 5.23 g/L and ethanol of 0.67 g/L, which shows similar results compared to that using glucose as the carbon source (Fig. 2c). The highest amount of acetic and butyric acids generated from starch could reach 2.40 and 4.21 g/L. On the other hand, when strain GL11 was cultivated in 60 g/L of xylan-amended medium, it could readily secret 1.82 U/mL of xylanase to hydrolyze xylan. However, as a more complex polysaccharide than starch, lower amount of xylose (2.45 g/L) occurred in the medium. After 144 h of fermentation, higher amount of ethanol (5.12 g/L) than butanol (1.48 g/L) produced. Meanwhile, higher amount of VFAs (2.08 g/L of acetate and 2.21 g/L of butyrate) were detected. The direct conversion of starch and xylan to butanol implied that strain GL11 could show potential for production of butanol from starchy-based material, such as cassava or food waste and lignocellulosic wastes.

## Discussion

In this study, the newly identified *C. pasteurianum* GL11 shows the highest butanol production titer and yield from glycerol compared to so far reported *Clostridium* strains (Table 2) and efficient direct conversion of polysaccharides (xylan and starch) to butanol, which are supported by (1) generating 14.7 g/L of butanol from glycerol with a yield of 0.41 g/g, surpassing the theoretical yield when using glucose; (2) integration with in situ extraction using biodiesel could further improve butanol to 28.8 g/L in the fed-batch mode and this sole butanol-enriched biodiesel can be directly used in biofuel sector; and (3) direct butanol and ethanol production from 60 g/L of starch (5.23 and 0.67 g/L) or xylan (1.48 and 5.12 g/L) via CBP. More importantly, the elimination of acetone and 1,3-PDO metabolic pathway within *C. pasteurianum* GL11 could further reduce the subsequent cost of separation. These metabolic properties of strain GL11 and process design can greatly improve the economic viability of biobutanol production both in terms of the associated substrate costs, by-products, and the downstream separation complexities.

**Fig. 6:**
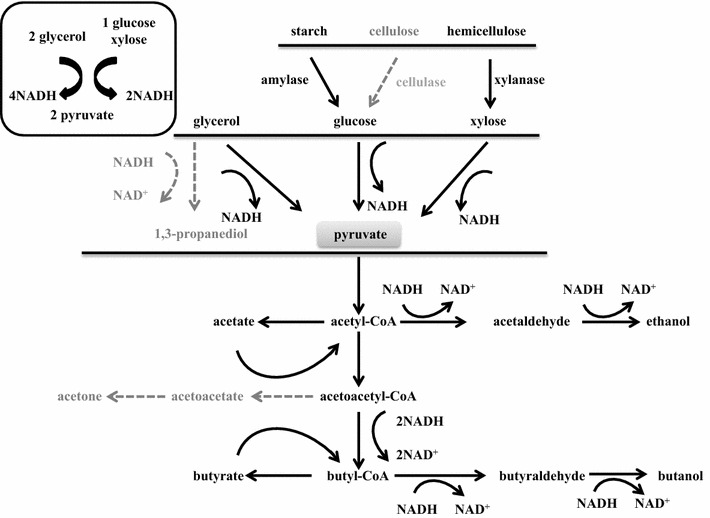
Metabolic pathway for BE production within *C. pasteurianum* GL11. *Dotted arrows* indicate deletion of the pathway

Glycerol is a cheaper, simpler, and more abundant molecule than glucose, which can be taken up into the microbial cells by facilitated diffusion, and a number of microorganisms possess metabolic pathways that can convert glycerol into different metabolic intermediates [5]. Currently, *C. pasteurianum* is the only known native producer of butanol by exclusive glycerol fermentation. However, due to the limitation of butanol tolerance, the butanol titer of most of reported *C. pasteurianum* strains is below 13 g/L [8, 9]. The newly isolated *C. pasteurianum* GL11 shows higher butanol production (14.7 g/L) in the batch and (28.8 g/L) fed-batch modes with in situ extraction. This could be because of its high butanol tolerance (up to 15 g/L) and efficient enzymatic expression when using glycerol as the substrate (Fig. 2e). Studies have shown that improvement of butanol tolerance within *C. pasteurianum* DSM 525 by chemical mutagenesis could significantly enhance the final butanol titer and yield from glycerol [9]. In addition, highly reduced nature of glycerol results in the generation of more reducing equivalents when it is converted to glycolytic intermediates such as pyruvate compared with glucose or xylose [25]. As shown in Fig. 6, 2 more NADH was produced when converted to 2 molecules of pyruvate than glucose or xylose. Besides, elimination of 1,3-PDO could save more NADH for butanol production compared to other *C. pasteurianum* strains [26, 27]. Hence, a subsequent result could theoretically be higher product yields than those obtained when glucose or xylose was used in the fermentation process by *C. pasteurianum* GL11.

The existence of by-products (such as, acetone, 1,3-PDO) not only impacts the economics of butanol fermentation, but also increases the separation costs [4]. Therefore, the engineering strategies for improving butanol selectivity, namely converting ABE fermentation into a single-butanol fermentation, have attracted general interests. However, several reports suggested that elimination of acetone generation metabolic pathways was often associated with an undesirable decrease in overall butanol titer due to the complexity of clostridia metabolic nets. Currently, no single-butanol-generating *Clostridium* strains have been constructed. Jiang et al. have disrupted acetoacetate decarboxylase gene (adc) in the hyper butanol-producing industrial strain *C. acetobutylicum* EA 2018 [18]. Although acetone formation was reduced to negligible amount (0.21 g/L), the butanol production was also dramatically decreased to only 7.4 g/L. The butanol yield was only increased by the addition of 6 mg/L of methyl viologen and 1 % calcium carbonate, which were used to alter the carbon flux and regulate the pH; however, this will further increase the whole economical costs [18]. Hence, wild-type *C. pasteurianum* GL11 with indigenous deficiency of acetone formation pathway and also high butanol production will show more advantages than those metabolically constructed strains through metabolic strategies. Besides acetone, 1,3-PDO is another main product when using glycerol as the substrate, which is essential for the balance of the redox within the cell. *C. pasteurianum* DSM 525 is even able to generate almost equal amount of 1,3-PDO and butanol from glycerol [9]. The genomic analysis of strain GL11 shows that both acetone and 1,3-PDO pathways are indigenously deficient due to the lackage of *adc* and *dhaT* genes. Further enzymatic assays also confirmed these findings. The elimination of by-products of acetone and 1,3-PDO when using glycerol as the substrate contributes to the high butanol yield in strain GL11 than other *C. pasteurianum* strains. Hence, the only BE-generating strain GL11 will provide another microbial platform for construction of a single-butanol-generating strain, which could be achieved by disruption of ethanol fermentation pathway.

CBP has been widely considered to be the best solution for cost-effective hydrolysis and fermentation of lignocellulosic biomass to biofuels or biochemicals [28]. Unlike most of solventogenic *Clostridium* species, strain GL11 shows capability of direct butanol and ethanol production from polysaccharides, such as xylan, representing another reported wild-type solventogenic *Clostridium* species which could directly convert xylan to butanol [28]. The success of butanol production via CBP was attributed to its efficient secretion of polysaccharides-degrading enzymes, like xylanase and amylase and further conversion of component sugars (glucose and xylose) in xylan and starch into biobutanol (Figs. 5, 6). Although butanol and total solvent production is higher than that in previous studies, where only 1.25 g/L of butanol occurred with elimination of ethanol from xylan by *Clostridium* sp. strain G117, which is the first reported solventogenic *Clostridium* strain for direct conversion of xylan to butanol [28], the butanol production was still much lower as compared to those using mono-substrates as the carbon sources, and more ethanol and VFAs were produced (Fig. 2c, d). This may be due to the following: (1) the low level of enzymatic xylan hydrolysis leading to insufficient formation of the reducing sugar necessary for butanol production [29] and (2) two more reducing factors of NADH are needed for butanol formation by re-assimilation of butyric acid (Fig. 6). Petitdemange et al. have also demonstrated that low reducing sugar concentrations in the fermentation medium resulted in only acid formation rather than solvent formation [29, 30]. Further studies are still needed to improve butanol titer via accelerating hemicelluloses degradation.

## Conclusions

This study presents how a newly identified *C. pasteurianum* GL11 can be used as a potential candidate for direct conversion of glycerol and polysaccharides into butanol. The wild-type strain GL11 was found to be particularly advantageous due to its capability of efficient butanol production from glycerol and polysaccharides with elimination of acetone and 1,3-PDO formation. Such observations can be considered highly promising for process commercialization where significant efforts need to be directed toward removal of unwanted solvents from the butanol following fermentation. The findings in this study thus offer fundamental knowledge for the future development of economically viable alternative fuel production strategies.

## Methods

### Isolation and phylogenetic analysis of strain GL11

Soils on the grassland in Shandong, China were collected as a source to screen for butanol-generating and xylan-degrading bacteria. At 35 °C and a pH of 6.5, bacterial community was anaerobically enriched by using mineral salts medium with xylan (10 g/L) as the sole carbon source. After several transfers, colonies on agar plates were selected when showing xylanolytic activity as indicated by Congo red staining method [30]. The xylanase activity of each colony was determined by measuring the zone of clearance on the agar plates. Butanol production was then investigated with glycerol as the substrate. One colony with butanol generation and superior xylan degradation capability, named GL11, was ultimately selected for the following investigation and phylogenetic identification based on 16S rRNA gene sequence [31–33]. Unless stated otherwise, the strain was anaerobically grown in mineral salts medium with xylan (10 g/L) as the sole carbon source and at its optimal conditions (30 °C, pH 7.0). The mineral salts medium contained the following: (g/L) NaCl 1.0, MgCl_2_·H_2_O 0.5, KH_2_PO_4_ 0.2, NH_4_Cl 0.3, KCl 0.3, CaCl_2_·H_2_O 0.015; (mg/L) FeCl_2_·4H_2_O 1.5, CoCl_2_·6H_2_O 0.19, MnCl_2_·4H_2_O 0.1, ZnCl_2_ 0.07, Na_2_MoO_4_·2H_2_O 0.036, NiCl_2_·6H_2_O 0.024, Na_2_WO_4_·2H_2_O 0.008, Na_2_SeO_3_·5H_2_O 0.006, H_3_BO_3_ 0.006, CuCl_2_·2H_2_O 0.002, 10 mM TES as buffer, and 5 g/L of yeast extract as nitrogen source.

### DNA extraction, genome sequencing, ORF prediction, and annotation

Cells of *C. pasteurianum* GL11 were collected from 10 mL culture by centrifugation at 10,000*g* for 10 min, then the pellet was washed by sterilized TE buffer twice to remove the residual medium before DNA extraction. The genomic DNA of *C. pasteurianum* GL11 was extracted by using a Qiagen genomic DNA kit with genomic-tip process (Qiagen, Germany), and verified to be high quality (DNA amount: ≥20 µg and purity: 1.8 ≤ OD_260nm/280nm_ ≤ 2.0).

The genomic DNA was firstly sheared randomly into fragments by Covaris S/E210 bioruptor for DNA fragment library preparation. After the desired fragments were received, a 500-bp paired-end library was constructed for sequencing using high-throughput Illumina sequencing technology with an Illumina HiSeq 2000 sequencer (Illumina Inc.). The paired-end reads were assembled by using SOAPdenovo (version 1.05), and assembly errors were corrected by using SOAPaligner (version 2.21). After obtaining the draft genome sequence, open reading frames (ORFs) were identified by using Glimmer (version 3.02), and the putative protein-coding sequences (CDSs) were functionally annotated by a series of reference databases, including GenBank, UniProtKB/TrEMBL, KEGG (Kyoto Encyclopedia of Genes and Genomes), COG (Clusters of Orthologous Groups), and UniProtKB/Swiss-Prot databases (identity threshold > 40 %). Genes for tRNA and rRNA were identified by tRNAscan-SE (Version: 1.21) and rRNAmmer (Version: 1.2), respectively. Searches for key enzymes involved in acetone and 1,3-PDO formation were performed based on the BLASTP.

### Fermentation and butanol tolerance experiments

Fermentation studies were conducted in 125 mL screw-capped bottles containing 100 mL of P2 medium. Prior to autoclaving the medium, the pH was adjusted to 6.0 using 2 M NaOH. The medium containing carbon source and yeast extract (5 g/L; Sigma, USA) was sterilized at 121 °C for 15 min. On cooling to 35 °C under oxygen-free nitrogen atmosphere (in an anaerobic chamber), filter-sterilized P2 stock solutions [(buffer: KH_2_PO_4_, 50 g/L; K_2_HPO_4_, 50 g/L; ammonium acetate, 220 g/L), (vitamin: para-amino-benzoic acid, 0.1 g/L; thiamin, 0.1 g/L; biotin, 0.001 g/L), and (mineral: MgSO_4_·7H_2_O, 20 g/L; MnSO_4_·H_2_O, 1 g/L; FeSO_4_·7H_2_O, 1 g/L; NaCl, 1 g/L)] were added (1 mL each), followed by inoculation with highly active cells of *C. pasteurianum* GL11 (5 mL cell suspension in 100 mL medium).

When pre-culture OD_600_ of 1.0 ± 0.05 was achieved (approximately 18 h), each culture was added to different amounts of butanol to achieve the concentrations of 5, 10, 15, and 18 g/L butanol. The growth and final butanol concentration in the presence of different concentrations of butanol were further monitored.

### Enzymatic assays

The activities of butanol dehydrogenase (BDH) were measured by monitoring NADH consumption at 365 nm according to the method described before [34] with some modifications. Cells were collected from 100 mL fermentation broth by centrifugation at 10,000*g* for 10 min using tightly sealed centrifuge tubes purged by nitrogen gas. The cell pellet was washed with Tris–HCl buffer (0.1 M, pH 7.5) once and resuspended in 5 mL the same Tris–HCl buffer. Lysis was carried out using a French press with one passage at 77 MPa. Supernatant was collected by centrifugation at 15,000*g* for 10 min and used for enzyme activity assay. Enzyme activity was calculated on the basis of a molar NADH extinction coefficient of 3.4 cm^−1^ mM^−1^. One unit of enzyme activity was defined as the amount of enzyme which converts 1 µmol NADH per minute under the reaction conditions. Protein concentration in cell extract was determined using the Bio-Rad protein assay kit with bovine serum albumin as a standard.

Xylanase and amylase activities were measured according to Bailey et al. [35] when serial enzyme dilutions were amended with 1 % (w/v) birchwood xylan or starch, 0.05 M glycine/NaOH buffer (pH 8.0). This mixture was incubated at 50 °C for 10 min. Reducing sugars released from xylanase or amylase were measured by using the 3,5-dinitrosalicylic acid (DNS) method [36]. One international unit (U) of each enzyme was defined as the enzymatic activity required for the release of 1 µmol xylose or glucose equivalents per unit volume and minute of reaction.

### Analytic method

Fermentation broth samples were analyzed for biomass growth, glycerol or sugar utilization, butanol, and solvent concentration. Biomass was determined by measuring optical density at 600 nm with appropriate dilution using a UV–visible spectrophotometer (Lambda-25, Perkin-Elmer, USA). 1,3-PDO, glycerol, glucose, and xylose were analyzed by a 1200 Series HPLC system (Agilent Technologies Inc.) equipped with an Aminex HPX-87H column (Bio-Rad, Richmond, CA, USA) and a Refractive Index Detector (RID). The samples were run at 75 °C with 0.6 mL/min eluent of 5 mM sulfuric acid. Biosolvents (i.e., acetone, ethanol, and butanol) and acids (i.e., acetic acid and butyric acid) were measured by a 7890 A gas chromatography (Agilent Technologies, USA) on a Durabond (DB)-WAXetr column (30 m × 0.25 mm × 0.25 µm; J&W, USA) equipped with a flame ionization detector (FID). The oven temperature was initially held at 60 °C for 2 min, increased to 230 °C at 15 °C/min, and held for 1.7 min. Helium was used as the carrier gas, with a flow rate of 1.5 mL/min. Five-point standard curves were obtained by running standard solutions containing biosolvents and acids.

### Nucleotide sequence accession number

The draft sequence data of *C. pasteurianum* GL11 are deposited at DDBJ/EMBL/GenBank databases under an Accession Number MCGV00000000. The version described in this paper is the first version. The full length of 16S rRNA gene of *C. pasteurianum* GL11 is deposited at GenBank with an Accession Number KX378861.

## Abbreviations

CBP: consolidated bioprocessing
ABE: acetone–butanol–ethanol
BE: butanol–ethanol
1,3-PDO: 1,3-propanediol
